# A correspondence on efficacy and safety of stem cell therapy in patients with dilated cardiomyopathy

**DOI:** 10.1097/JS9.0000000000001743

**Published:** 2024-05-28

**Authors:** Leisheng Zhang, Hongju Yang, Jing Li

**Affiliations:** a Science and Technology Innovation Center, The Fourth People’s Hospital of Jinan (The Teaching Hospital of Shandong First Medical University); b Department of Oncology, The Fourth People’s Hospital of Jinan City, The Teaching Hospital of Shandong First Medical University, Jinan; c Geriatric Medical Center, Division of Geriatric Gastroenterology, The First Affiliated Hospital of Kunming Medical University, Kunming; d National Health Commission (NHC) Key Laboratory of Diagnosis and Therapy of Gastrointestinal Tumor, Gansu Provincial Hospital, Lanzhou, People’s Republic of China

*Dear Editor,*

The recently published article entitled ‘Efficacy and safety of stem cell therapy in patients with dilated cardiomyopathy: an umbrella review of systematic reviews’ has captured our attention^1^. This article reviewed prior literature to find the meta-analysis and systematic review that met inclusion criteria and conducted a summary analysis of these studies. The review suggests that stem cell therapy (SCT) holds promise in treating dilated cardiomyopathy (DCM), with many studies highlighting its safety and potential benefits. However, there are some questions that we need to discuss.

Firstly, various outcomes such as Major Adverse Cardiac and Cerebrovascular Events (MACCE) in Table 2, All-cause Mortality, and Health-Related Quality of Life (HRQL) were presented in one of the included reviews. It is possible that certain outcomes reported across multiple studies were overlooked.

Secondly, while most of the reviews reported that the type of intervention was bone marrow cell (BMC), the nearly published review by Diaz-Navarro *et al*.^2^ in 2021 included 13 randomized controlled trials (RCTs) and another review by Wang *et al*.^3^ included 20 RCTs in 2019. It is concerning why there is such a significant discrepancy in the number of included RCTs between the two reviews, especially considering that the review by Wang *et al*. was conducted earlier than the review by Diaz-Navarro *et al*.

Thirdly, in the published systematic reviews of RCTs on the topic of stem cell therapy in patients with dilated cardiomyopathy, it would be prudent to conduct an updated meta-analysis that includes the highest number of RCTs with a more comprehensive search strategy. The umbrella review is limited by these and other problems.

## Ethical approval

Our submitted manuscript does not involve any patients without the ethical approval document.

## Consent

Our submitted manuscript does not involve any patients without the written informed consent documents.

## Source of funding

None.

## Author contribution

L.Z. and H.Y.: participated in the original idea and writing the first draft; J.L.: edited the final manuscript. All authors approved the last version of this manuscript.

## Conflicts of interest disclosure

There are no conflicts of interest among the authors.

## Research registration unique identifying number (UIN)

None.

## Guarantor

Leisheng Zhang.

## Data availability statement

The supporting data used in the paper can be obtained via the requirement from the authors.

## Provenance and peer review

Commentary, internally reviewed.

## References

[1] Ran J Dziedzic A Naser IH . Efficacy and safety of stem cell therapy in patients with dilated cardiomyopathy: an umbrella review of systematic reviews. Int J Surg (London, Engl) 2024. [Epub ahead of print]. doi:10.1097/js9.0000000000001142 PMC11487037 38320100

[2] Diaz-Navarro R Urrútia G Cleland JG . Stem cell therapy for dilated cardiomyopathy. Cochrane Database Syst Rev 2021;7:CD013433. 34286511 10.1002/14651858.CD013433.pub2 PMC8406792

[3] Wang C Li J Zhang B . Safety and efficacy of bone marrow-derived cells therapy on cardiomyopathy: a meta-analysis. Stem Cell Res Ther 2019;10:137. 31109372 10.1186/s13287-019-1238-5 PMC6528271

